# Real-Time Detection of Sleep Apnea Based on Breathing Sounds and Prediction Reinforcement Using Home Noises: Algorithm Development and Validation

**DOI:** 10.2196/44818

**Published:** 2023-02-22

**Authors:** Vu Linh Le, Daewoo Kim, Eunsung Cho, Hyeryung Jang, Roben Delos Reyes, Hyunggug Kim, Dongheon Lee, In-Young Yoon, Joonki Hong, Jeong-Whun Kim

**Affiliations:** 1 ASLEEP Inc Seoul Republic of Korea; 2 Department of Artificial Intelligence Dongguk University Seoul Republic of Korea; 3 Department of Psychiatry Seoul National University Bundang Hospital Seongnam Republic of Korea; 4 Seoul National University College of Medicine Seoul Republic of Korea; 5 Department of Otorhinolaryngology Seoul National University Bundang Hospital Seongnam-si Republic of Korea

**Keywords:** sleep apnea, OSA detection, home care, artificial intelligence, deep learning, prediction model, audio, diagnostic, home technology, sound

## Abstract

**Background:**

Multinight monitoring can be helpful for the diagnosis and management of obstructive sleep apnea (OSA). For this purpose, it is necessary to be able to detect OSA in real time in a noisy home environment. Sound-based OSA assessment holds great potential since it can be integrated with smartphones to provide full noncontact monitoring of OSA at home.

**Objective:**

The purpose of this study is to develop a predictive model that can detect OSA in real time, even in a home environment where various noises exist.

**Methods:**

This study included 1018 polysomnography (PSG) audio data sets, 297 smartphone audio data sets synced with PSG, and a home noise data set containing 22,500 noises to train the model to predict breathing events, such as apneas and hypopneas, based on breathing sounds that occur during sleep. The whole breathing sound of each night was divided into 30-second epochs and labeled as “apnea,” “hypopnea,” or “no-event,” and the home noises were used to make the model robust to a noisy home environment. The performance of the prediction model was assessed using epoch-by-epoch prediction accuracy and OSA severity classification based on the apnea-hypopnea index (AHI).

**Results:**

Epoch-by-epoch OSA event detection showed an accuracy of 86% and a macro F_1_-score of 0.75 for the 3-class OSA event detection task. The model had an accuracy of 92% for “no-event,” 84% for “apnea,” and 51% for “hypopnea.” Most misclassifications were made for “hypopnea,” with 15% and 34% of “hypopnea” being wrongly predicted as “apnea” and “no-event,” respectively. The sensitivity and specificity of the OSA severity classification (AHI≥15) were 0.85 and 0.84, respectively.

**Conclusions:**

Our study presents a real-time epoch-by-epoch OSA detector that works in a variety of noisy home environments. Based on this, additional research is needed to verify the usefulness of various multinight monitoring and real-time diagnostic technologies in the home environment.

## Introduction

Multinight prescreening and follow-up monitoring of obstructive sleep apnea (OSA) at home might lead to better management [1]. For instance, if multiple prescreening of sleep disordered breathing can be performed at home in a simpler way, OSA underdiagnosis can be improved by motivating patients to visit the hospital. In addition, if OSA can be monitored frequently at home during weight loss for OSA treatment, it will motivate patients to make more active efforts. Since OSA occurs during sleep, diagnosis and monitoring of sleep breathing disorders require objective tests. Attended polysomnography (PSG) and the home sleep apnea test (HSAT) with a reduced number of sensor channels have been playing an essential role in the diagnosis of OSA. However, due to their high cost and accessibility, multinight prescreening or follow-up monitoring using conventional sleep tests (levels I, II, III, and IV), such as PSG and HSAT, is difficult. Furthermore, successful assessment via PSG comes at a cost of burdensome sensor equipment and manual sleep scoring by clinicians. Beyond conventional sleep tests, many efforts have been made to achieve more convenience, fewer sensors, and even noncontact assessment of OSA [2]. Sound-based OSA assessment holds great potential since it can be integrated with smartphones and smart speakers to provide full noncontact monitoring of OSA.

The nature of breathing sounds during sleep varies according to the patency of airways. During sleep, as the activity of the upper airway dilator muscle decreases, the collapsibility of the upper airway increases. Thus, the breathing sound might become louder than that during wakefulness. When an apnea event occurs, no breath sound can be heard due to a cessation of breathing. However, when the apnea is over, the airway reopens and a loud breath sound can be produced. As for hypopnea, unlike snoring, the airway is narrowed without airway vibration. Thus, it can be estimated that the breathing sound will become smaller and irregular. Therefore, it is expected that it will be possible to detect respiratory events based on sounds generated during sleep. Many studies have been conducted on the acoustic characteristics of breathing sounds [3,4]. Since these studies focused on the acoustic features of ordinary breathing sound, the entire-night nocturnal sound was given as input and the OSA severity of that night was evaluated. However, a more natural and correct way to diagnose OSA is to detect and count individual apneic and hypopneic events. Only a few recent studies have tried to detect apnea events by observing the recovery breath or loud gasp after an apnea event [5]. Deeper investigations into OSA event detection under various and severe home environment noises are needed.

Acoustic apnea detectors validated in hospitals might fail at home as they can be affected by various types of noises from a residential environment. A machine learning or deep learning model for in-home assessment should be trained and tested with data collected from a home sleep study to guarantee performance. However, the difficulty of a home sleep study is well known. Similar difficulties have been challenged in various deep learning fields, such as speech recognition [6]. To train a robust deep learning model in real-world speech applications, clean speech data were contaminated [6]. While adding noises, such as alarms, door knocks, telephone ringing, and television, the signal-to-noise ratio (SNR) can be randomly set to generate abundant combinations of distorted sounds.

In this study, we aim to build a real-time detector of sleep apneas and hypopneas based on breathing sounds recorded on a smartphone during sleep and the prediction will be reinforced by training with home noises.

## Methods

### Sleep Breathing Sound Data Set

We included a total of 1315 audios with full-night, in-laboratory PSG performed between January 2015 and December 2020. Among them, 1018 (77.41%) recordings were from the PSG microphone (SUPR-102, ShenZhen YIANDA Electronics Co. Ltd., Shenzhen, China) installed on the ceiling 1.7 m above the subject’s head, and 297 (22.59%) recordings were from the smartphone microphone (LG G3, LG Electronics, Inc, Seoul, Republic of Korea) placed on a bed table 1 m away from the subject. Some recordings were made simultaneously with both microphones. However, such recordings were excluded from this study for subject-independent evaluation.

We split our sleep breathing data set into a training data set and a test data set. The test data set was composed of only smartphone audios since our region of interest was at-home service. The test data set was designed to have a similar number of subjects for AHI<15 events/hour and AHI≥15 events/hour, since 15 was often used as a threshold to decide the prevalence of problematic OSA. Detailed baseline subject characteristics of the test data set are presented in Table 1. The rest of the data belonged to the training data set. Although PSG audios accounted for the majority of the training data set, they provided sufficient information to train the model due to their similarity to smartphone audios with a large number and variety of data.

**Table 1:** Demographic characteristics of subjects in the sleep breathing data set (N = 1315).

Characteristics		Training and validity (n=1165, 88.59%), n (%)	Test (n=150, 11.41%), n (%)
Age (years), mean (SD)		52.77 (13.70)	47.14 (12.36)
Males, n (%)		825 (70.82)	125 (83.33)
**BMI**			
	BMI, mean (SD)	26.02 (4.09)	26.51 (3.99)
	BMI<18.5, n (%)	22 (1.89)	2 (1.33)
	18.5≤BMI<25, n (%)	513 (44.03)	52 (34.67)
	25≤BMI<30, n (%)	467 (40.09)	76 (50.67)
	BMI≥30, n (%)	163 (13.99)	20 (13.33)
**AHI^a^**			
	AHI, mean (SD)	24.84 (23.55)	24.11 (19.42)
	AHI<5, n (%)	255 (21.88)	27 (18.00)
	5≤AHI<15, n (%)	268 (23.00)	48 (32.00)
	15≤AHI<30, n (%)	268 (23.00)	48 (32.00)
	AHI≥30, n (%)	374 (32.10)	27 (18.00)

^a^AHI: apnea-hypopnea index.

### Home Noise Data Set

The home noise data set consisted of various types of sounds that might occur in a residential environment. A total of 22,500 sounds were downloaded from Freesound [7], an open sound effect library. The length of the sounds ranged from 30 seconds to 2 hours. The home noise data set was grouped into 9 different categories: home appliance, room noise, contents, clock, speech, animal, air conditioner and fan, rain and wind, and car and motorbike (see Table S1 in Multimedia Appendix 1). Each group was divided into training noises and test noises. Training noises were used along with the training data set in consistency training for model robustness. Test noises were added to the test data set to simulate sleep breathing sounds recorded in various home environments.

### Preprocessing

In this study, the OSA event presence detection window was set as 1 epoch (30 seconds). The entire night recording of each patient was divided into 30-second segments and converted to a Mel spectrogram for visual representation of audio to visualize how the sound energy in each frequency bin changed over time. The Mel spectrograms were synchronized with manually annotated sleep apnea events from PSG. In this paper, we omitted central apnea and regarded only OSA events as apnea.

Each Mel spectrogram was labeled as one of “apnea,” “hypopnea,” or “no-event” according to the presence or absence of an apnea event during the segment period. If there was no event during the segment period, the Mel spectrogram was labeled as “no-event.” If there was 1 existing event, the Mel spectrogram was labeled following the type of event. If 2 types of events existed in 1 segment period, the segment was labeled as the type of event having a longer period.

### Training Overview

The acoustic apnea event detector model inputs Mel spectrograms of sleep breathing sounds and outputs the distribution probability of the OSA event classes for each epoch (“apnea,” “hypopnea,” or “no-event”). In this work, the confidence of a class in a prediction refers to its probability given by the model. The event class that had the highest confidence was chosen to be the final estimation of the model for the input. The overnight AHI was estimated based on the epoch-by-epoch detection result. The algorithm for training the model was divided into 2 components. The first component was supervised learning for OSA event detection. For supervised learning, the model was trained to predict the existence of apnea events from the input of sound data using a large-size data set. The second component was home noise consistency training. Home noises were used to simulate home sleep sounds by adding them to sleep breathing sounds, and we trained the model with simulated home training data sets to make the model robust. Testing with a clean test data set validated our model in a hospital environment. The model was also tested with various simulated home environments generated by adding various noises with different SNRs.

### Deep Neural Network Architecture

In this work, we modified the SoundSleepNet [8] architecture originally designed for sleep stage prediction, for epoch-by-epoch OSA event detection. Although predicting the sleep stage requires long-term analysis of how the respiratory pattern and breathing sound change, detecting OSA events does not require such long-term analysis. Since the length of apneic events mostly ranges from 10 to 60 seconds, observing the past and future for just 1 or 2 epochs will help. Although the original architecture processed 40 Mel spectrograms to output sleep stages of the middle 20 epochs (40-20), we modified the architecture to get input of 14 epochs and output 10 epochs of OSA event labels (14-10). Here, the OSA event label is one of “apnea,” “hypopnea,” and “no-event.” In detail, the model architecture was composed of 2 core elements: feature extractor and multiepoch detector. The feature extractor observed each Mel spectrogram to extract features of unique sounds of apneic events. Then, the multiepoch detector located epochs that contained apneic events, and predicted the type of the events by analyzing neighboring features.

### Overcoming Class Imbalance With Class Weights

To overcome the problem of data imbalance among classes, where the amount of data in the “no-event” class dominated those of other classes, we used different weights for different classes. To make sure the model did not get too biased to “no-event,” we penalized the incorrect predictions of “apnea” and “hypopnea” more by giving higher weights to them than to “no-event” during training. By empirical methods, we found assigning the weights of 1.0, 1.3, and 2.1 to “no-event,” “apnea,” and “hypopnea,” respectively, gave the best result.

### Home Noise Consistency Training

Consistency loss is a key to making the model work under home noise. It guides the model to output similar predictions with and without the noise, which means the model becomes robust to the noise. Consistency loss is defined as a mean-squared error (MSE) between the prediction of the clean sleep breathing sound and the prediction of the corrupted version of that sound. To generate a corrupted sound, noise data were randomly sampled from training noises and added to the clean sleep breathing sound with random SNRs ranging from –20 to 5. Since the length of the input sequence was 14 epochs, which was 7 minutes in time, we sampled noise until the total length of the sampled noise reached more than 7 minutes. The concatenated noise was then added to the input sequence to generate the corrupted sound.

**Figure 1:**
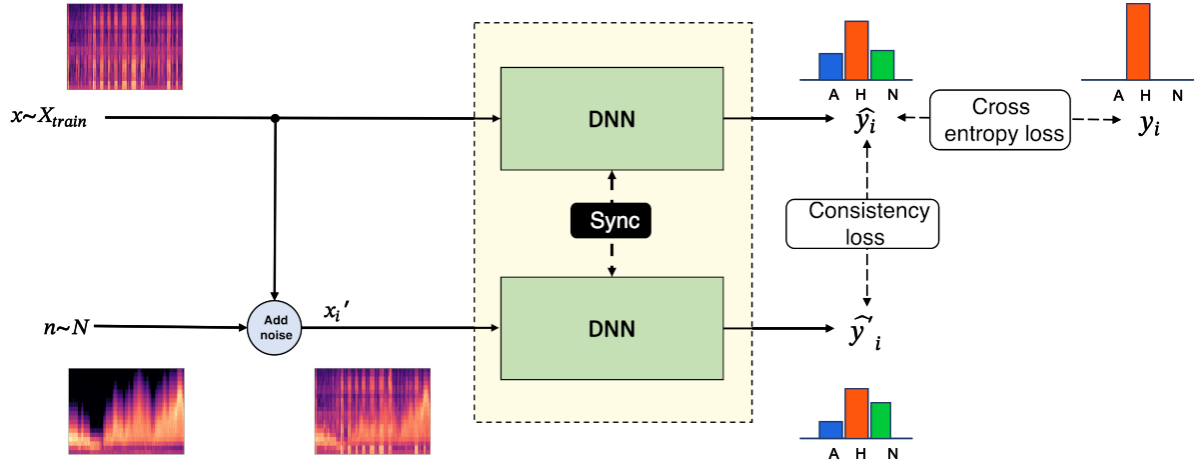
Consistency training with noise-added inputs. In each training iteration, 1 input sequence of the Mel spectrogram, 1 sequence of noise, and 1 SNR value between –20 and 5 are chosen. The noise sequence is added to the clean input with respect to the given SNR to make the noise-added version of the clean input. Both clean and noise-added inputs are fed into the model 1 by 1. Two loss terms are calculated at this stage: (1) the cross-entropy loss between the output from the clean input data and the ground truth and (2) the consistency loss between the output from clean and noise-added inputs. The final loss is the summation of the 2 loss terms. It is used to update the model accordingly. DNN: deep neural network; SNR: signal-to-noise ratio.

The weighted sum of consistency loss and cross-entropy loss was used to train the model. The cross-entropy loss, which is generally used for supervised training, represents the difference between the predicted apnea class probability and the observed label. The cross-entropy loss can be calculated only from the clean sleep breathing sound data. It guides the deep learning model to predict the correct class. A simple data augmentation (pitch shifting) was applied for additional robustness. Figure 1 illustrates the consistency training process

We adopted a 2-step training algorithm: (1) 1-to-1 pretraining and (2) 14-to-10 training [8]. In the 1-to-1 pretraining, where the model worked with 1 epoch data, we used an initial learning rate of 0.01 and decreased it 10 times whenever the macro *F*_1_-score in validation did not increase for 3 consecutive epochs. As for the 14-to-10 training, the primary model, the slanted triangular learning rate scheduler, and gradual unfreezing were applied for better fine-tuning of the pretrained model [9]. For the detailed training setting, we used the stochastic gradient descent optimizer and fixed the number of training epochs at 10 in all experiments. The checkpoint with the highest macro *F*_1_-score in the validation was selected as the best model.

### Statistical Analysis

#### AHI Estimation With Regression Modeling

Since OSA events are annotated independently of the sleep stage label, a single event can be split into 2 or 3 consecutive epochs. On the contrary, 2 short events can belong to 1 epoch. Thus, regression modeling was performed to estimate the AHI from the average counts of estimated apneic epochs per hour. Statistics showed that a linear function model successfully represented the relationship between the number of epochs that were labeled as “apnea/hypopnea” and the AHI reference value from PSG for the data in the training data set. Thus, we adopted the linear regression model to estimate the AHI value, which is simple and fast enough to be used in real-time applications. The best linear fit with the RANSAC regressor [10] was derived from the training data set. It was used to estimate the AHI value during the test. See Multimedia Appendix 1 for details.

#### Evaluation Metrics

We evaluated our model on 2 aspects: epoch-by-epoch OSA event detection and overnight AHI estimation. For epoch-by-epoch OSA event detection, we evaluated the model for the 3-class case (“apnea,” “hypopnea,” and “no-event”) and the 2-class case (“apnea/hypopnea” and “no-event”). The performance was evaluated using several metrics (ie, accuracy, Cohen κ, and macro *F*_1_-score). We selected the macro *F*_1_-score as our primary metric since it is known to be less sensitive to class imbalance problems. For overnight AHI estimation, the model was evaluated mainly with sensitivity, specificity, and the area under the curve (AUC) of OSA screening, with AHI cut-offs of 5, 15, and 30, respectively. Moreover, AHI estimation results were directly compared with the observed AHI using a Bland-Altman plot.

### Ethical Considerations

The use of the Hospital PSG dataset in this study was approved by the Institutional Review Board (IRB) of SNUBH (IRB No. B-2011/ 648-102). As per the Smartphone PSG dataset, a written informed consents were obtained according to the Declaration of Helsinki and the study protocol was approved by the Institutional Review Board of Seoul National University Bundang Hospital (IRB No. B-1912-580-305). All participants signed the written consents before the recording were made.

## Results

### Performance of the Model in a Hospital Environment

#### Epoch-by-Epoch OSA Event Detection

**Figure 2:**
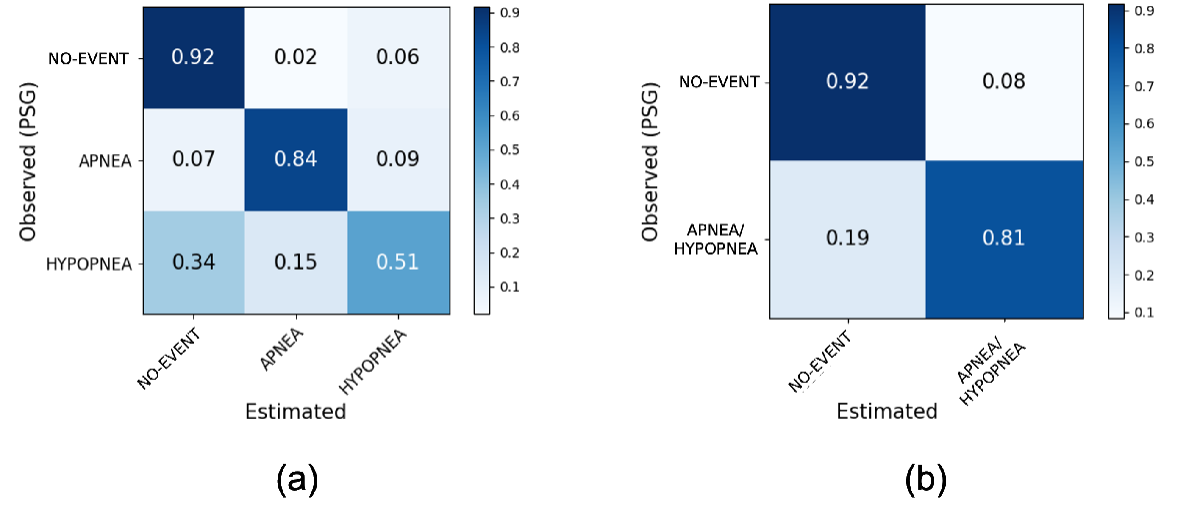
Epoch-by-epoch confusion matrices of the model's detection for (a) 3-class (“apnea,” “hypopnea,” “no-event”) and (b) 2-class (“apnea/hypopnea” and “no-event”) classifications. PSG: polysomnography.

In this section, the model was evaluated with a clean test data set collected in a hospital environment. In the 3-class case, the accuracy was 86% and the macro *F*_1_-score was 0.75. The model had an accuracy of 92% for “no-event,” 84% for “apnea,” and 51% for “hypopnea.” Most misclassifications were made for “hypopnea,” with 15% and 34% of “hypopnea” being wrongly predicted as “apnea” and “no-event,” respectively (Figure 2). In a 2-class case where “apnea” and “hypopnea” were merged into 1 class, the model achieved an accuracy 88.8%, with a macro *F*_1_-score of 0.86 (Table 2).

**Table 2:** Epoch-by-epoch OSA^a^ event detection performance of the model with a clean data set without noise (the model takes 30-second epochs as input and outputs the classification result of the corresponding epoch for 3- and 2-class cases).

Classification and parameters		Value
**3-Class cases (“apnea,” “hypopnea,” and “no-event”)**		
	Macro *F*_1_-score	0.75
	Accuracy (%)	85.9
	Cohen κ	0.66
	“No-event” sensitivity	0.92
	“No-event” specificity	0.81
	“Apnea” sensitivity	0.84
	“Apnea” specificity	0.96
	“Hypopnea” sensitivity	0.52
	“Hypopnea” specificity	0.93
**2-Class cases (“apnea/hypopnea” and “no-event”)**		
	Macro *F*_1_-score	0.86
	Accuracy (%)	88.8
	Cohen κ	0.71
	“Apnea/hypopnea” sensitivity	0.81
	“Apnea/hypopnea” specificity	0.92

^a^OSA: obstructive sleep apnea.

**Figure 3:**
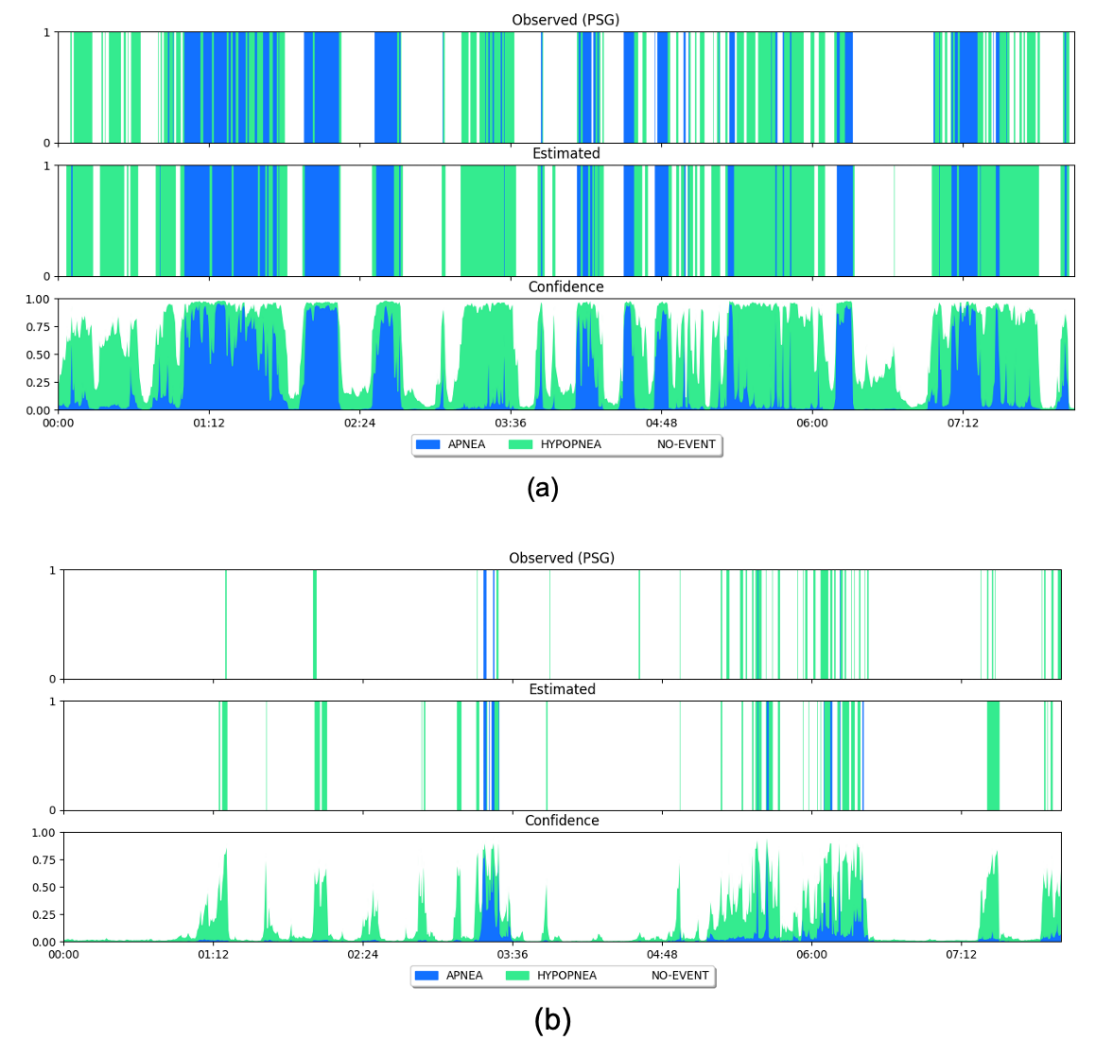
Epoch-by-epoch comparison between observed events from PSG (first row) and predicted results using our method (second row) for 2 subjects: (a) AHI=70.1 and (b) AHI=9.2. Each vertical line represents the label for one 30-second epoch over the whole night. The last row presents probabilities of distribution over 3 classes predicted by our model, which indicates the confidence of model prediction. AHI: apnea-hypopnea index; PSG: polysomnography.

Figure 3 displays 2 examples that compare observed events from PSG and predicted events using our method for 2 subjects. One is in the severe-sleep-apnea group (Figure 3a; AHI reference value=70.1 events/hour, macro *F*_1_-score=0.81), and the other is in the mild-sleep-apnea group (Figure 3b; AHI reference value=9.2 events/hour, macro *F*_1_-score=0.70). The model output predictive probabilities of each of the 3 classes, called “confidence,” as shown in the last row of Figure 3. From the whole data set, the average confidence of the correctly predicted epochs was 0.89 and the average confidence for the incorrectly predicted epoch was 0.67, meaning that the model is more confident when it predicts correctly. Similar trends were observed for the 2 patients with severe and mild apnea, respectively. For the first subject, the average confidence was 0.80 for correctly predicted epochs and 0.65 for incorrectly predicted epochs. The average confidence for the second subject was 0.87 for correctly predicted epochs and 0.59 for incorrectly predicted epochs.

#### Overnight AHI Estimation

Table 3 summarizes the performance of OSA risk screening assessed with the binary classification with generally accepted AHI cut-off points (5, 15, and 30 events/hour). The model achieved the performance with an AHI cut-off of 5, showing a sensitivity, specificity, and AUC of 0.97, 0.89, and 0.93, respectively. With an AHI cut-off value of 15, the sensitivity, specificity, and AUC were 0.85, 0.84, and 0.85, respectively. With an AHI cut-off value of 30, the model had a sensitivity, specificity, and AUC of 0.96, 0.91, and 0.94, respectively.

**Table 3:** Overnight AHI^a^ estimation. Performance results of the model, including sensitivity, specificity, and AUC^b^, are presented, with the number of nights below and above each AHI cut-off point.

AHI cut-off points “c”	5	15	30
Number of subjects AHI<c, n (%)/AHI≥c, n (%)	27 (18)/123 (82)	75 (50)/75 (50)	123 (82)/27 (18)
Sensitivity	0.97	0.85	0.96
Specificity	0.89	0.84	0.91
AUC	0.93	0.85	0.94

^a^AHI: apnea-hypopnea index.

^b^AUC: area under the curve.

**Figure 4:**
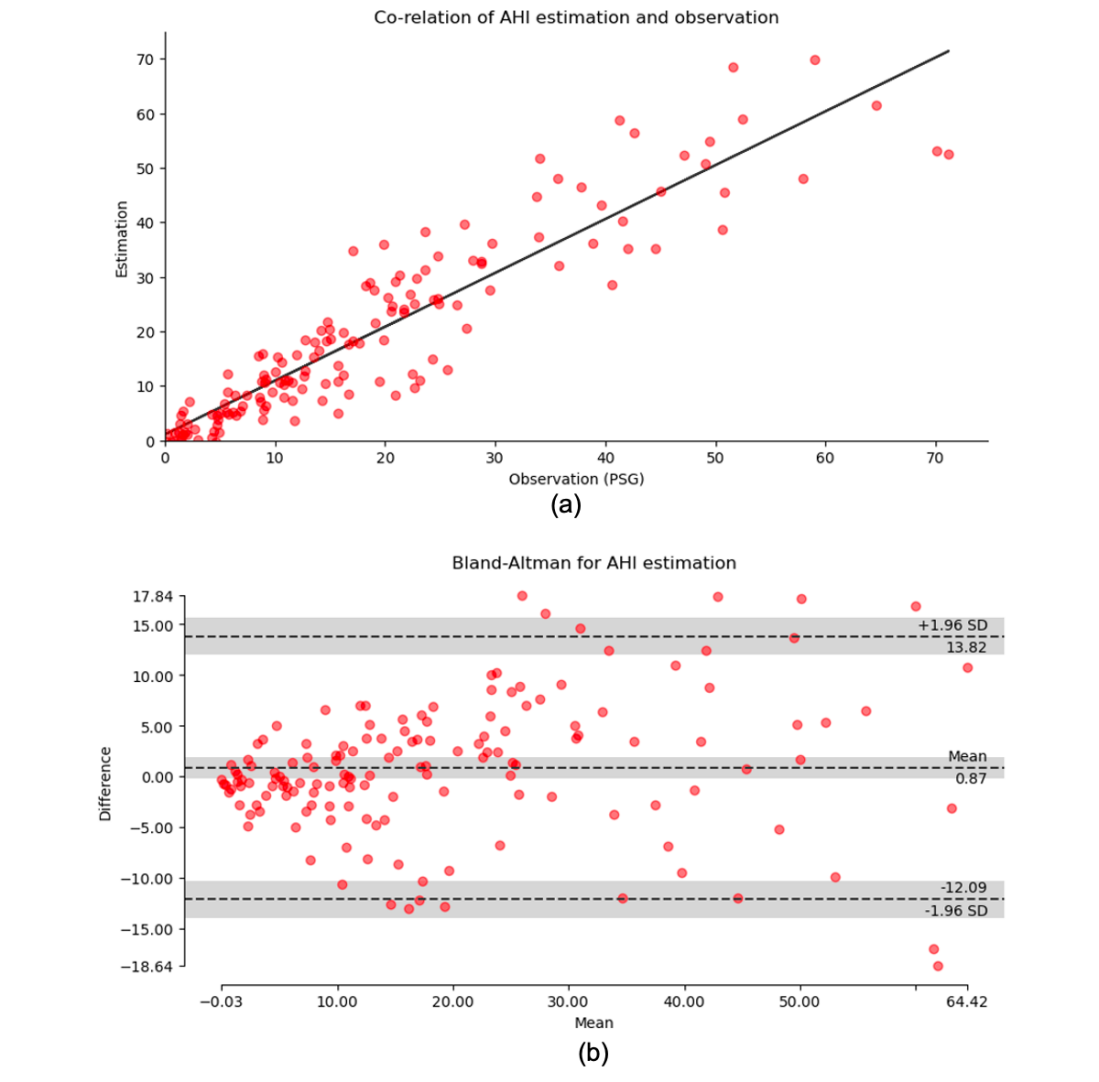
Overnight AHI estimation. (a) Correlation and (b) Bland-Altman plots of estimated and measured AHI. AHI: apnea-hypopnea index; PSG: polysomnography.

Figure 4a shows a correlation plot between the observed AHI and AHI estimates. It shows a mean absolute error of 4.88 events/hour and a correlation coefficient of 0.98. Figure 4b displays a Bland-Altman plot. The mean difference between the AHI estimation and the observed value was approximately 0.87 (95% CI −12.09 to 13.82) events/hour.

### Performance of the Model in a Simulated Home Environment

One of the key goals of this research was to validate the model in a simulated home environment by adding various types of home noises to a clean test data set. Experimental results showed an effect of the proposed training method with home noise and verified the robustness of our model in a noisy environment.

#### Robustness to Noise With Varying SNRs

Table 4 shows the results of the comparison between our model and a control model. The control model adopted the same neural network architecture as ours. However, it was trained only with clean sleep sound data. Both models were tested using the home noise–added test data set with varying SNRs (5 to –30). As the SNR decreased (the power of the noise sound became larger), the performances of both models degraded, with the degradation of our model being relatively smaller than that of the control model. When the power of the noise and sleep sound was the same, that is, SNR=0, our model had a macro *F*_1_-score of 0.72, which showed a 4% degradation compared to testing with a clean test data set, whereas the control model had a macro *F*_1_-score of 0.64, with a 15% degradation.

**Table 4:** Epoch-by-epoch comparison of the macro F^1^-score between our model and the control model across multiple SNR^a^ values (10 to –30).

SNR	No noise	5	0	–5	–10	–15	–20	–25	–30
Macro *F*_1_-score of our model	0.75	0.73	0.72	0.71	0.69	0.68	0.65	0.62	0.58
Macro *F*_1_-score of the control model	0.75	0.67	0.64	0.61	0.57	0.52	0.47	0.43	0.39

^a^SNR: signal-to-noise ratio.

#### Impact of the Noise Group on Training and Testing

**Figure 5:**
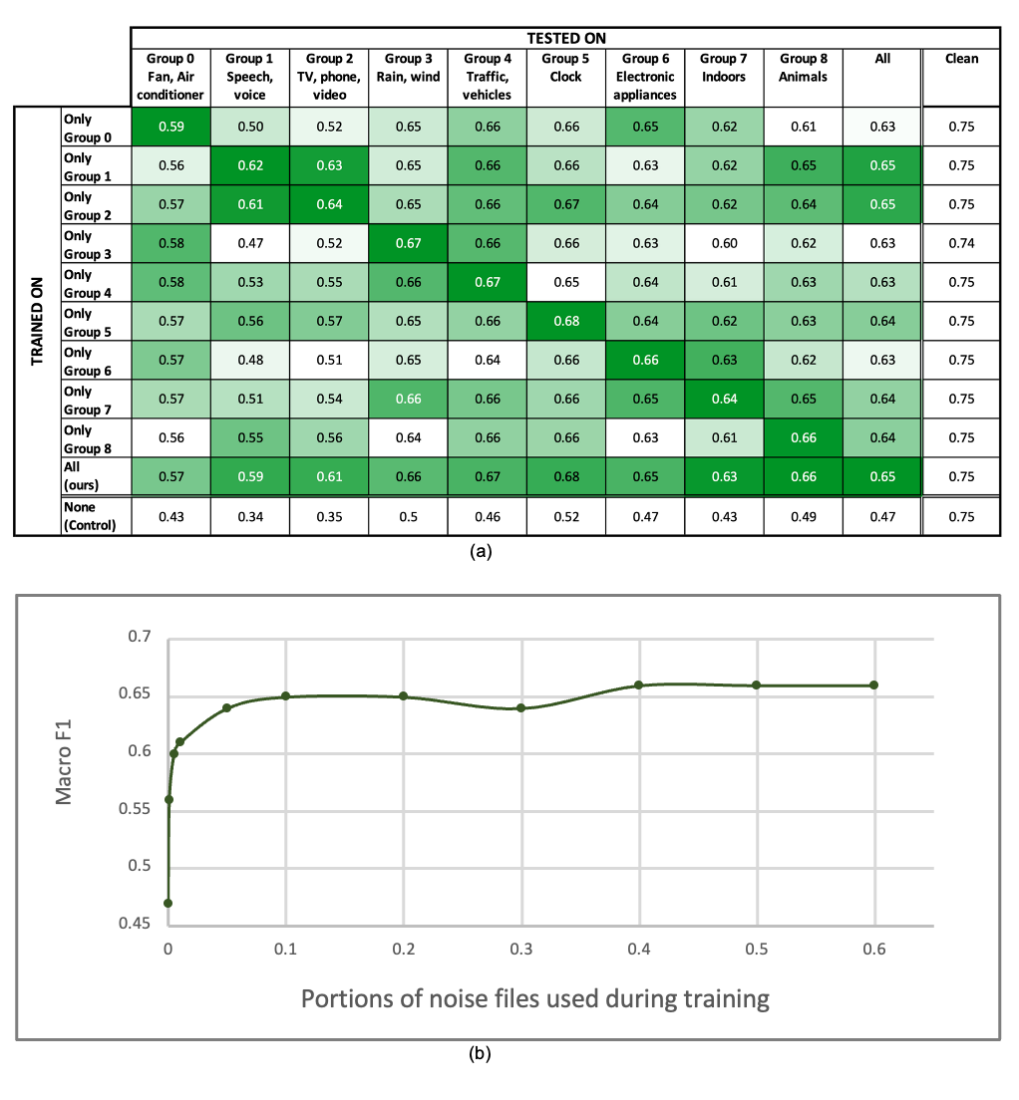
Epoch-by-epoch results for the impact of training with noises on performance. (a) The result of 1 group training and testing for 9 different noise groups. Each row of the first 9 rows represents a model trained on the same train data set, with additional noise from 1 noise group. The columns represent the test data sets that the models were tested on. We created a heat map based on the value in each column: The highest value is presented in dark green, while the lowest value is presented in white. For better visualization, we left all the test on clean data (rightmost column) and from the control model (bottom row) uncolored. (b) Performance of the model with different portions of noise files used during training.

Figure 5a presents the effects of different noise types. The home noise data set was grouped into 9 different categories according to the noise source. Detailed information about the noise grouping can be found in Multimedia Appendix 1. We trained 9 different models. Each model used only 1 noise group for training. Each trained model was validated with 9 test data sets simulated by adding each noise group with an SNR of –50.

We observed that our model was weak for certain noise groups, such as fan and air conditioner (group 0), human voice (group 1), and digital content (group 2). The macro *F*_1_-score of the model dropped to 0.57, 0.59, and 0.61 for groups 0, 1, and 2, respectively. There was a performance gap between the control model and our model. It was significant for groups 1 and 2 when the model was tested with test data sets simulated with noise related to human speech. Training with simulated home sound improved the macro *F*_1_-score from 0.34 to 0.59-0.61 for groups 1 and 2. Each model achieved the best macro *F*_1_-score when trained and tested with the same noise group, explaining the bolder diagonal in Figure 5a. Our model trained with all noise groups achieved the best or second-best performance when tested with each noise group 1 by 1 and performed best on the test data set combined with all noise groups.

Figure 5b shows the effects of the number of noise files used while training. As more noise files were used for training, the performance of the model improved. However, it was almost saturated at near 4500 files, which accounted for 20% of our home noise data.

**Figure 6:**
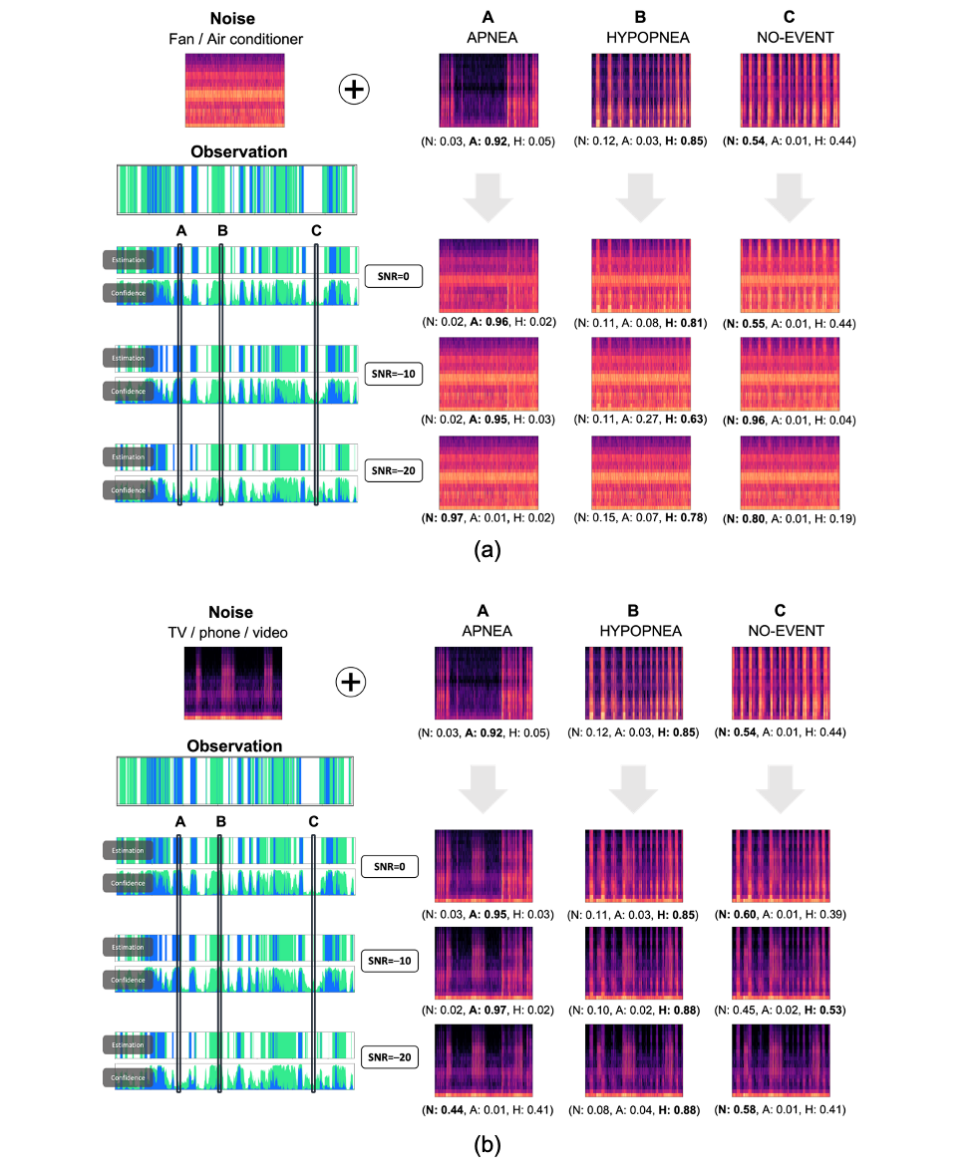
Epoch-by-epoch effects of noise types and SNR values on the model’s prediction for the same sleep epochs. One noise file was selected representing either (a) stationary or (b) event-driven type of noise. The file was added into the same select time frames, each from 1 class (denoted as A, B, and C). The top row displays Mel spectrograms of the clean data. Each row below represents Mel spectrograms of each noise-added data with respect to the SNR values written in the middle of the figure and the clean data above. Estimations and confidence graphs of the model corresponding to each value of SNR are displayed on the right-hand side of the figure. SNR: signal-to-noise ratio.

Figure 6 presents a Mel spectrogram of 3 epochs (“apnea,” “hypopnea,” and “no-event”). The “no-event” epoch showed a regular pattern of breathing sounds, while “apnea” had a recovery breath or a loud gasp after events. Figure 6a shows the Mel spectrogram corrupted by noise with an SNR of 30, which makes it hard for a human to detect OSA. However, even in this case, the model succeeded in classifying all events with high confidence. The result also showed the effects of different types of noise. As shown in Figure 6a, when a stationary noise was added, the model was robust for “apnea” and “no-event” epochs but not for “hypopnea.” However, as shown in Figure 6b, when an event-driven noise was added, an opposite trend was found.

## Discussion

### Principal Findings

This study proposed a robust acoustic apnea event detector and proved that it could work under a simulated home environment generated with various groups of background noises and nonstationary noises from the interior and exterior. Our key findings were as follows:

- The proposed model could detect the sound of a recovery breath or a loud gasp after an apnea event. Therefore, it could successfully detect epoch-by-epoch OSA events.
- By leveraging noises during model training, the model was able to overcome the simulated noisy environment in the test.
- The proposed model was robust for various noise types that the model never experienced during the training.

Therefore, the model trained with a limited number of noises can work well for a general environment with various noises.

### Effect of Home Noise–Added Sleep Breathing Sounds

To the best of our knowledge, this is the first work to use simulated PSG-labeled home-recorded sounds generated by simply adding separately collected home noises to in-lab sleep breathing sounds to train and test the model for OSA event detection. Benefits of simulated sounds are as follows: First, simulated sounds are indistinguishable from the real sounds recorded in a home environment, because the characteristics of the sounds are additive. Second, we can generate various intended sounds by controlling the SNR and types of noises. In addition, sounds that rarely occur in realistic home environments or even sleep sounds where humans can hardly fall asleep can also be generated. Finally, both home noises and in-lab sleep breathing sounds are easy to collect.

Experiment results proved the impact of using the simulated home-recorded sounds during training. First, our model seemed to overcome a noisy environment where the control model failed to distinguish noise from the sound of apneic events. The performance of the control model dropped rapidly even from SNR=5, where the average power of noise was less than one-third of that of the sleep breathing sound. However, the performance of our model was maintained until SNR=–5, where the average power of noise was more than 3 times that of the sleep breathing sound. The performance gap between the control model and our model kept increasing when the power of noise increased until the SNR was –30.

Second, our model was able to be generalized to inexperienced noise. When trained on only 1 group of noise at a time, the model showed an acceptable performance degradation of 3% on the macro *F*_1_-score, even with the lowest SNR setting of –20. This indicates that our model will also work on unseen types of noises in practice. Moreover, the model reached saturated performance even with only 20% of the entire noise. This means that a limited but enough number of noises could make the model become generalized enough to work with any noises.

Experimental results demonstrated that our model is weak for certain types of noise. For the stationary type of noise that does not change over time, such as fan and air conditioner noises, the sleep breathing sound is masked, making it more difficult to detect the event. The noise group experiment showed a similar trend. The model showed worse performance for fan and air conditioner (group 0) noises known to have a typical stationary noise than for other noise groups. Other noise groups for which our model (trained with noise) and the control model (trained without noise) did not work well were groups 1 and 2, which were related to human speech. The performance degradation was more severe for the control model because in the training data that recorded the sound during PSG, the speech might be recorded when subjects were awake and OSA labels of these epochs were always “no-event.” Thus, the model tended to predict the epoch as “no-event” when speech was included in the input sound. However, this was alleviated when we applied the proposed training by adding noise.

### Comparison With Other Studies With Contact Biosignals

We compared our study with other studies examining various biosignals other than sounds, such as pulse oximetry, electrocardiography (ECG), and pressure sensors. Several studies have used pulse oximetry as an input to the model [11-13]. In a recent study [11] of automatic OSA detection based on pulse oximetry analysis with 92 subjects, the epoch-by-epoch 2-class macro *F*_1_-score was 0.84, with an accuracy of 91%. These performance results were similar to those of our model. That recent study [11] showed a higher accuracy but a lower macro *F*_1_-score than ours. The study also estimated an AHI value through similar counting and regression modeling as ours. In that study [11], for OSA risk screening with an AHI cut-off of 15 events/hour, the sensitivity was 0.96 and the specificity was 0.95, which outperformed our model because oxygen desaturation was a concomitant of incomplete breathing. The ECG is another signal that can be used for AHI estimation [14,15]. A study [14] with 97 subjects achieved a sensitivity of 0.89 and a specificity of 0.83 when the AHI cut-off was 10. One study [16] used more than 1 signal simultaneously, such as a pressure sensor signal, sound, actigraphy, and respiration. It reported a sensitivity of 0.88 and a specificity of 0.89 at an AHI cut-off of 15 with 118 subjects [16]. These performance results were comparable to or better than ours. However, unlike the study that required a contact device to measure the signal or buy an additional device [16], ours is easier to implement because breathing sounds can be easily recorded by a smartphone.

### Comparison With Other Sound-Based Studies

Several studies have taken nocturnal sound as an input source. One study extracted features of the sound for the whole night and applied machine learning algorithms to those features to directly estimate the overnight AHI value [4] and achieved a sensitivity of 0.87 and a specificity of 0.71, with an AHI cut-off of 15 using 167 subjects. A recent study [17] took the Mel spectrogram for an epoch (30-40 seconds) as an input to the model and estimated the AHI through regression modeling. For OSA risk screening with a cut-off of 15, it achieved a sensitivity of 0.79 and a specificity of 0.80 with 103 participants [17]. These studies focused only on the estimation of the overnight AHI. However, our model not only outperformed these other models in estimating the AHI value but also provided information about which events might occur and when using epoch-by-epoch OSA event detection.

### Application

One of the key contributions of this study is that our model is designed for epoch-by-epoch detection of OSA events in real time. Therefore, it can be used along with real-time intervention devices, such as light and sound intervention, vibration, and position change, to improve respiratory disturbances during sleep at home [17,18]. In addition, our model provides convenient detection of OSA events overnight and allows for easier long-term multinight monitoring of patients with sleep apnea in the home environment. Thus, this method can also be used to monitor the effect of lifestyle modification.

### Limitation of This Work

Results suggest that our model works well in distinguishing “no-event” and “apnea,” while the performance for classifying “hypopnea” is not as good. Reasons can be twofold: (1) Data imbalance among the 3 classes is severe in the real world, where the number of samples for hypopnea is merely one-tenth of that for the “no-event” class, and (2) clues for detecting apnea are strong enough that the model can predict even in a noisy environment, while irregular patterns in hypopnea can be mistaken for noise from the environment. Even with a carefully calibrated set of class weights, the problem of data imbalance is fundamentally difficult and can only be solved optimally by gathering enough data. In future studies, we will use a more diverse data set using the data we have been collecting with local hospitals. Further collection of data and building a better model could solve this problem. Merging with other devices for different biosignals might improve the performance of the model for detecting hypopnea.

### Conclusion

In conclusion, this study presents a smartphone-based acoustic apnea event detector with potential to be used as a tool for multinight prescreening and follow-up monitoring of OSA at home. By leveraging home noises during model training and testing, we built a model proven to be robust in various home environments. A deeper analysis gained more insight into conditions under which the model could practically be used. Future works are needed to use epoch-by-epoch OSA event detection for real-time intervention devices to mitigate the severity of OSA.

## Appendix

Multimedia Appendix 1 Details of the AHI estimation regression method and noise groups used. AHI: apnea-hypopnea index.

## Abbreviations

AHI: apnea-hypopnea index
AUC: area under the curve
HSAT: home sleep apnea test
OSA: obstructive sleep apnea
PSG: polysomnography
SNR: signal-to-noise ratio
