# Supplementary material for: Real-Time Detection of Sleep Apnea Based on Breathing Sounds and Prediction Reinforcement Using Home Noises: Algorithm Development and Validation
Source: J Med Internet Res. 2023 Feb 22;25:e44818. doi: 10.2196/44818 (PMC9996414; doi:10.2196/44818)
Supplement: Multimedia Appendix 1 [file jmir_v25i1e44818_app1.docx]

## Supplementary Materials

### Regression model for overnight AHI estimation


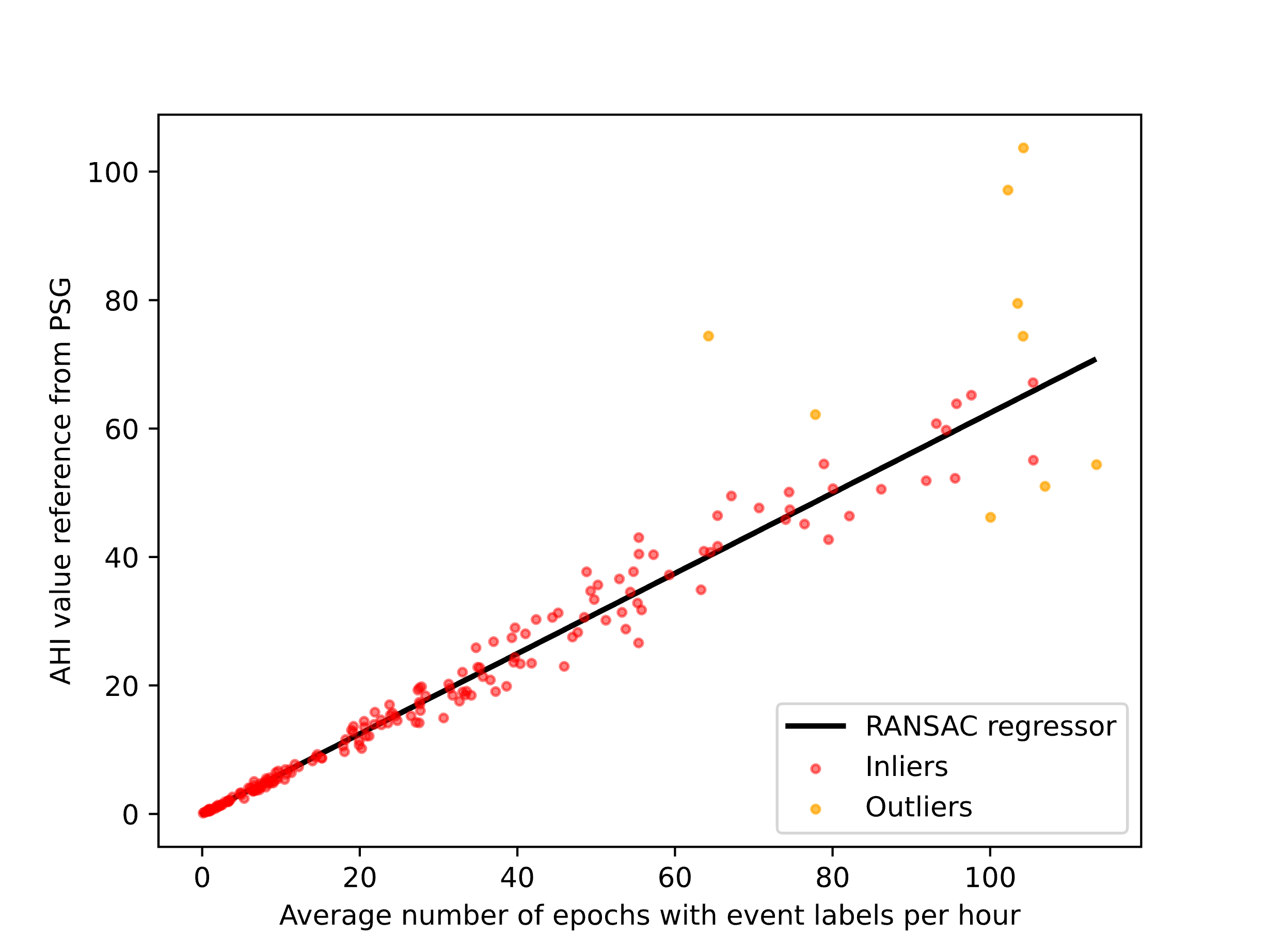


Figure S1. Relationship between AHI reference values from PSG and ratios of the number epoch with estimated events to the total time for the training set.

We modeled the formula to obtain the estimated AHI values as follow:

$$\hat{AHI}=\alpha\times\frac{\text{No. of epochs with events}}{\text{Total no. of epochs}}+\beta$$

By fitting the model to the distribution of our train dataset, we obtained empirical values of $\alpha=0.63$, and $\beta=-0.18$. This model was used to estimate the AHI value from model’s predictions in validation and test stages.

### Divide noise groups

We first downloaded the various types of sound that might occur in the residential environment. We defined the groups of noise using the keywords that are most likely to appear in the tags of the noise audio in the group. Using the API provided by FreeSound, we queried the noise files of each group based on the tags defined from the previous step. The audio files were filtered to be longer than 30 seconds and have user rating above 4.0. We then carefully filtered the list of downloaded noise files to remove strange audio by removing files containing unrealistic tags. The remaining audio files were then converted into Mel-spectrograms of 30 seconds epochs. Finally, we limited the number of epochs in each group for balancing during training and testing processes.

Table S1. Statistic of noise groups

| Group | Included noise types | | Quantity (epochs) |
| --- | --- | --- | --- |
| 0 | Fan, Air-conditioner | | 2,500 |
| 1 | Speech, voice | | 2,500 |
| 2 | TV, phone, video | | 2,500 |
| 3 | Rain, wind | | 2,500 |
| 4 | Vehicles, traffics | | 2,500 |
| 5 | Clocks | | 2,500 |
| 6 | Electronic appliance | | 2,500 |
| 7 | Indoors | | 2,500 |
| 8 | Animals | | 2,500 |
|  | | Total | 22,500 |
